# Extramedullary hematopoiesis in cancer

**DOI:** 10.1038/s12276-024-01192-4

**Published:** 2024-03-05

**Authors:** Derek A. G. Barisas, Kyunghee Choi

**Affiliations:** Department of Pathology and Immunology, Washington University School of Medicine, St. Louis, MO USA

**Keywords:** Haematopoietic stem cells, Tumour immunology, Tumour immunology, Translational immunology, Myelopoiesis

## Abstract

Hematopoiesis can occur outside of the bone marrow during inflammatory stress to increase the production of primarily myeloid cells at extramedullary sites; this process is known as extramedullary hematopoiesis (EMH). As observed in a broad range of hematologic and nonhematologic diseases, EMH is now recognized for its important contributions to solid tumor pathology and prognosis. To initiate EMH, hematopoietic stem cells (HSCs) are mobilized from the bone marrow into the circulation and to extramedullary sites such as the spleen and liver. At these sites, HSCs primarily produce a pathological subset of myeloid cells that contributes to tumor pathology. The EMH HSC niche, which is distinct from the bone marrow HSC niche, is beginning to be characterized. The important cytokines that likely contribute to initiating and maintaining the EMH niche are KIT ligands, CXCL12, G-CSF, IL-1 family members, LIF, TNFα, and CXCR2. Further study of the role of EMH may offer valuable insights into emergency hematopoiesis and therapeutic approaches against cancer. Exciting future directions for the study of EMH include identifying common and distinct EMH mechanisms in cancer, infectious diseases, and chronic autoimmune diseases to control these conditions.

## Exploring EMH: The Future of Emergency Hematopoiesis and Cancer Therapy

Changes to hematopoiesis, or the creation of blood and immune cells, in cancers are the focus of this review by Barisas et al. They discuss conditions that promote extramedullary hematopoiesis (EMH), which produces blood cells outside the bone marrow. They also discuss the impact of tumor-derived factors, including interleukin-1α and leukemia inhibitory factor, on EMH. The team recently discovered that these specific factors stimulate the development of myeloid cells known for suppressing the immune system and aiding tumor growth. This suggests that targeting the pathways associated with these elements could be a new approach to cancer treatment. Research also indicates that EMH might be a common characteristic in other inflammation-related diseases, providing unique opportunities for further studies.

This summary was initially drafted using artificial intelligence, then revised and fact-checked by the author.

## Introduction

Hematopoiesis is the continuous process by which blood and immune cells are produced by the actions of hematopoietic stem cells (HSCs). Thought to be organized similarly to an elevated water source draining into a branching system of rivers, HSCs can continue to self-renew and differentiate to produce mature differentiated cells, including red blood cells, T-cell lineages, B-cell lineages, monocyte lineages, and neutrophils. A critical component of hematopoiesis is the niche that regulates HSC self-renewal and differentiation, which is crucial for hematopoietic output. The niche includes both hematopoietic and nonhematopoietic lineages that perform unique but sometimes overlapping roles. While occurring primarily in the bone marrow of adult animals, hematopoiesis can occur in extramedullary sites during times of organismal stress to increase or sustain hematopoietic output, a phenomenon known as extramedullary hematopoiesis (EMH). In cancer, EMH is increasingly recognized as a mechanism by which cancer cells generate a favorable immune environment for growth. For example, tumor cells can utilize EMH to produce immunosuppressive hematopoietic subsets.

Cancer is a major health concern in the United States of America and globally. In the United States, cancer is the second leading cause of death and costs more to treat than any other disease^1^. Like other chronic inflammatory pathologies, including arthritis and myocardial infarction, solid tumors enhance the production of myeloid cells, termed myelopoiesis, to further their own growth at the expense of their host^2–4^. In part, this increased myelopoiesis leads to a high ratio of neutrophils to lymphocytes in the peripheral blood, which is correlated with poor survival in breast, colon, pancreatic, and gastric cancer patients and in a systematic review of all cancer types^5–8^. As immune-based cancer therapeutics become more widely used, attention has turned to modulating myeloid cells in the tumor microenvironment to improve their efficacy^9^. One potential avenue is to target their production at the extramedullary hematopoietic site. Here, we review the role of EMH in cancer and other inflammatory conditions and the proteinaceous factors contributing to EMH in adults. Moreover, we discuss the hematopoietic niche in various hematopoietic organs to deepen the understanding of the unique contributions of EMH to physiological and pathological outcomes.

## EMH in cancer

EMH refers to the expansion of blood cells in the extramedullary sites of a mature animal in response to an altered organismal state. EMH does not include hematopoiesis in organs such as the thymus at homeostasis and in any organ during development. There are three general processes that induce EMH: (1) trapping of proliferative hematopoietic progenitors in the spleen during hyposplenism; (2) impairment of hematopoietic capacity in the bone marrow due to damage or myelophthisis; and (3) abnormal levels of circulating factors with extramedullary hematopoietic capabilities^10^. Although the most common organs involved in EMH are the spleen, liver, and lymph nodes, organs as diverse as the skin, pleura, adrenal gland, and pancreas have demonstrated EMH activity^11–13^. For factor-induced EMH, hematopoiesis-active cytokines and pathogen-associated molecular patterns, including G-CSF, GM-CSF, IL-3, and IL-6:sIL-6R complexes, as well as lipopolysaccharide and Pam3CSK4, respectively, have been shown to play roles in stimulating EMH^14–17^. Some instances of EMH induced by these factors have marked effects. Subcutaneously injected human IL-3 was reported to induce cutaneous hematopoiesis with trilineage potential at the injection site in cynomolgus monkeys^17^. One patient was reported to have trilineage cutaneous hematopoiesis following G-CSF therapy to treat myelofibrosis^18^.

The role of EMH in cancer is a rapidly growing research focus. EMH is associated primarily with hematologic cancers but can also occur in patients with breast, lung, renal, colon, gastric, pancreatic, or prostate cancer^4,11,13,19^. In particular, splenic EMH has been recognized for more than 30 years in the context of human solid tumors with or without bone marrow metastases^20^. The presence of EMH is potentially important for cancer treatment because EMH preferentially induces the production of myeloid cells. In the context of cancer, increased myeloid cells are recognized as having a negative impact on survival. A high ratio of neutrophils to lymphocytes in the peripheral blood was correlated with poor prognosis in a systematic review of all cancer types, and in pancreatic, colon, breast, and gastric cancers individually^5–8^. In a case series on EMH, Bao et al.^11^ reported that breast cancer was the most common solid cancer reported with EMH, 7.1% of all patients had confirmed EMH in the spleen, and 24% of patients had splenomegaly, which is clinically associated with splenic EMH. The first mouse model of splenic EMH in the context of solid tumors was created by Cortez-Retamozo et al.^21^, in which the authors demonstrated that splenic myeloid progenitors contributed directly to the tumor environment and tumor growth and that human spleens similarly expanded myelopoietic progenitors during invasive solid tumor progression. Wu et al.^22^ suggested that increased circulating myelopoietic progenitors in patients with solid tumors contributed to suppressing myeloid cell generation and the formation of an immunosuppressive tumor microenvironment. Subsequently, Wu et al.^4^ reported increased human hematopoietic stem and progenitor cells (HSPCs) in the red pulp of the spleen in four separate solid tumors and demonstrated that gastric cancer patients with low levels of EMH in their red pulp have a more favorable prognosis. Additionally, these authors found that the splenic HSPCs generated in their mouse model of hepatocellular carcinoma were myeloid-biased and showed modifications to the splenic niche^4^. A more recent paper identified HSPCs within the tumor mass of human glioblastomas and revealed that their presence was associated with tumor grade^23^. We recently evaluated the role of EMH specific, myeloid-expanded HSPCs and their splenic niche in EMH initiation and maintenance^24^. Together, these data establish a series of novel and important discoveries about the presence and role of EMH in a diverse set of solid tumors. Furthermore, given the correlation between the increased neutrophil to lymphocyte ratio and poor prognosis in patients with solid tumors, targeted, prospective studies to identify EMH sites and EMH-inducing factors in patients with solid tumors and concomitant peripheral myeloid skewing may be justified.

EMH may not mimic all aspects of bone marrow hematopoiesis. EMH seems to occur in humans most commonly because of a loss of hematopoietic capacity in the bone marrow, in agreement with the observation that EMH occurs secondarily to hematologic malignancy^13^. When eliminating hepatosplenic hematopoiesis, the most common condition associated with EMH was myelofibrosis with myeloid metaplasia, and the most common location was the thoracic vertebral column^25^. Generally, EMH is associated with myeloid- or erythroid-biased differentiation, although extrathymic T-cell development has been reported in transgenic models^26^. The mobilization of HSPCs often coincides with the initiation of EMH and is important in seeding cells for this process. However, the extent to which the proliferation of local progenitors contributes to EMH is unknown. Additionally, the various factors that induce EMH begs the question of the degree of diversity that exists within the broad framework of EMH. Is EMH a single unified endpoint for multiple types of inflammation that broadly increase myelopoietic capacity, or do different forms of inflammation produce distinct types of EMH? Answering this question may shed light on the contribution of EMH to various disease states.

## EMH and myelopoiesis

Myelopoiesis, the production of myeloid lineage cells from HSPCs, is a process that occurs during homeostasis but is also highly responsive to the organismal state^27,28^. The development of myeloid cells involves a complex balance of select transcription factors throughout the differentiation process^29^. The transcription factors that favor myeloid differentiation include PU.1, CCAAT/enhancer binding proteins (C/EBPα, C/EBPβ and C/EBPε), growth factor independent 1 (GFI1), and interferon-regulatory factor 8 (IRF8)^30–35^. PU.1 is a crucial transcriptional regulator of myeloid differentiation, followed by IRF8, which delineates the monocyte lineage, and C/EBPα, which delineates the neutrophil lineage^30,31,35^. In times of organismal stress, the nature of factors driving myelopoietic differentiation can be altered. For instance, mice lacking *Cebpb* maintain normal granulocytopoiesis while in homeostasis but fail to induce emergency granulocytopoiesis during challenge^36^. Cytokine factors, including G-CSF, GM-CSF, M-CSF, IL-1, and IL-27, can stimulate the production of myeloid cells^37–41^.

The tumor microenvironment (TME) contains a diverse population of myeloid cells similar to that generated during homoeostasis, including neutrophils, macrophages, and dendritic cells, as well as subsets not present at homeostasis, such as myeloid-derived suppressor cells (MDSCs)^42–44^. This review will focus on granulocytic MDSCs (G-MDSCs) due to their association with solid tumors, the ongoing study of their developmental origin, and their presumed effects on treatment outcomes and prognosis. Compared with homeostatic neutrophil subsets, G-MDSCs exhibit positive CD84 expression in mice and humans and positive LOX1 expression in humans in addition to their functional capacity to inhibit T-, B-, and NK-cell activation^44–46^. It is important to note that many historical studies have not separated neutrophils from tumor-associated neutrophils or neutrophils from G-MDSCs due to their similar surface phenotype and the recent discovery of distinctive markers. Because G-MDSCs have been shown to be the most abundant neutrophil subset in the circulation and in the TME during late-stage tumor progression, unless the cited paper specifically distinguishes neutrophils in cancer from G-MDSCs, it is reasonable to assume that these neutrophils are indeed G-MDSCs.

In addition to the expansion of myelopoiesis, changes in the cytokine milieu also impact the cellular products of myelopoiesis, most prominently, the production of G-MDSCs^44^. Many factors are required for stimulating emergency granulocytopoiesis, and a secondary factor directs the polarization of G-MDSCs toward an immunoregulatory phenotype^47^. Two particularly important signaling pathways are the NF-κB and STAT1 and STAT3 pathways^48^. The factors implicated in inducing immunosuppressive activity through the NF-κB pathway include TNFα, IL-1β, and Toll-like receptor ligands, while IFNγ is most commonly linked to STAT1 activation^49,50^. G-CSF may be important for inducing STAT3 activation in developing G-MSDCs^51,52^. Interestingly, G-CSF has also been shown to promote the development of immunosuppressive neutrophils at the expense of dendritic cells capable of cancer immunosurveillance^53,54^. Together, these data implicate an extensive list of cytokines that could contribute to the pathological myelopoiesis identified in cancer. Future research should strive to identify the factors that initiate the cascade of myelopoiesis and the relative contributions of immune-derived and tumor-derived factors to the induction and maintenance of myelopoiesis. Additionally, these studies did not address the contribution of EMH to the induction of pathological myelopoiesis, and emerging data suggest that EMH is an important producer of pathological G-MDSCs in the context of solid tumors.

G-MDSCs are recruited into the tumor environment through the binding of their CXCR2 receptor to CXCL1, CXCL2, and CXCL8^55^. In keeping with its importance in the TME, high systemic CXCL8 levels in tumors increase the number of neutrophils, presumably G-MDSCs, and recruit cells to the tumor microenvironment, reducing the efficacy of anti-PD-L1 therapy^56,57^. Conversely, CXCR2 deletion or the use of anti-Ly6G antibodies in mouse models slows tumorigenesis^58–62^. Once in the TME, G-MDSCs maintain immunosuppression through the promotion of angiogenesis and metastasis and reduced responsiveness to immune checkpoint blockade^57,63,64^. To promote angiogenesis, G-MDSCs have been shown to express MMP9, BV8, and VEGF^65–68^. G-MDSCs have also been shown to promote metastasis by enhancing the epithelial–mesenchymal transition of tumor cells through the upregulation of hepatocyte growth factor and TGF-beta^69^. At the site of metastasis, G-MDSCs can capture circulating tumor cells through neutrophil extracellular traps and surface markers^59,70^. The presence of granulocytes with altered phenotypes in the presence of chronically inflamed, solid tumors has sparked investigations into the developmental processes that lead to their production and the crucial mechanistic processes that can be targeted for therapeutic intervention.

## Bone marrow hematopoietic niche

To understand how alterations in hematopoiesis occur during cancer, one must understand the hematopoietic niche of the bone marrow, as this region is the primary site of hematopoiesis during homeostasis. The bone marrow HSC niche exists in a complex microanatomical environment that fosters the differentiation and self-renewal of HSCs while also directing their response to organismal changes^71,72^. While many cell types have been implicated to play a role in this niche, here, we will focus on perivascular stromal cells, endothelial cells, sympathetic nerves, and macrophages. Prominent within the bone marrow hematopoietic niche are cell types known as perivascular stromal cells, mesenchymal stem cells, or osteoblast precursor cells, which will be collectively treated as the same cell type here, as recent evidence does not support their separation into distinct cell types. The expression of both CXCL12 and KIT ligands distinguishes these cells from other constituents of the bone marrow niche^73,74^. These mesenchymal stem cells in the bone marrow most commonly distinguished by leptin receptor, nestin, Mx1, Prx1, Osx, PDGFRα, or CD51 expression^75–77^. Initial studies also identified osteoblasts as critical components of the niche^78,79^. However, when CXCL12 or KIT ligands were deleted from mature osteoblasts, no significant changes in hematopoietic lineages were observed^74,77,80^. This finding likely reflects the fact that mesenchymal stem cells of the bone appear capable of differentiating into osteoblasts in vitro and were misconstrued as representing mature osteoblasts in early studies^81^. In fact, under proper culture conditions, mesenchymal stem cells are capable of maintaining HSCs in vitro^75,76^. Endothelial cells of the bone marrow contribute multiple factors that play a role in the bone marrow niche, including E-selectin, basic FGF, DLL1, IGFBP2, angiopoietin 1, DHH, and EGF^82–87^.

Within the bone marrow, the vascular niche is thought to be split into an arteriolar and a sinusoidal-megakaryocyte component. The arteriolar components were first identified as the preferential location of quiescent HSCs in the endosteal region of the bone marrow^88^. In addition to endothelial cells and mesenchymal stem cells, the arteriolar niche includes sympathetic neurons and nonmyelinating Schwann cells, each with their own niche contribution. Sympathetic neurons alter CXCL12, angiopoietin 1, KIT ligand, and VCAM-1 expression in mesenchymal stem cells through β3-adrenergic receptor signaling and thus enhance mobilization^89,90^. Schwann cells contribute to the activation of TGFβ, a regulator of HSC quiescence^91,92^. In addition to the endothelial and mesenchymal stem cell components, the venous sinusoidal niche also contains megakaryocytes that reduce HSC proliferation through CXCL4 and TGFβ but also promote recovery after radioablation through FGF1^93–96^. Macrophages also effect the bone marrow niche through their regulation of CXCL12 expression on mesenchymal stem cells^97,98^. The bone marrow niche also responds to a variety of signals, including circadian rhythms, prostaglandins, pathogen-associated molecular patterns, and hormones^89,99–102^. Ironically, in a model of primary myelofibrosis in the bone marrow niche, the overgrowth of mesenchymal stem cells reduces the amount of the marrow space available for hematopoietic cells^103^.

Additionally, the hematopoietic niche undergoes remodeling in response to myelopoietic stimuli during aging or in patients with obesity^104–108^. Phenotypes associated with aging can be rescued by altering sympathetic signaling within the bone marrow, indicating that sympathetic nervous system activity may play a part in age-related changes in hematopoiesis^106,107^. In obesity, the role of adipocytes in modulating the niche has been an important topic of study^109^. However, varying effects of adipocytes on HSPC maintenance and differentiation have been reported. Initial studies linked BM with high adiposity to lower hematopoietic output^110,111^. Other data have shown that BM adipose tissue is capable of producing important hematopoietic cytokines, such as KITL and CXCL12, while being able to support hematopoiesis in vitro^112–114^. Additionally, adipocytes are recognized as contributing myeloid-biasing cytokines such as TNFα and IL-6^115,116^. When taken together, the components of the bone marrow niche supply many factors, often in conflict with each other, that drive and alter continued hematopoietic function.

## Extramedullary hematopoietic niche

EMH is an important topic in clinical medicine and offers numerous opportunities to further our understanding of hematopoiesis itself. One area of interest is understanding the extramedullary niche as a unique tissue that does not necessarily mimic the bone marrow but nevertheless recapitulates the principal factors involved in hematopoietic development. However, the diverse niche components that are involved in hematopoiesis in nonbone marrow sites are poorly characterized. While several aspects of the splenic hematopoietic niche in adult animals have been studied, comparatively few studies have evaluated the liver, lymph node, or skin niche. In addition, EMH of the spleen is localized to sinusoids of the red pulp, where both mesenchymal stem cells and endothelial cells produce the KIT ligand and only mesenchymal stem cells produce CXCL12^117^. In a liver model of EMH, CXCL12 appeared to be upregulated in sinusoidal endothelial cells^118^. Moreover, KIT ligand and CXCL12 double-positive cells was present within the lesion of an adult patient with nodular, cutaneous EMH^119^.

Despite these similarities, tissue-specific differences have also been identified. Splenic mesenchymal stem cells are leptin receptor-negative and express Tlx1^120^. Additionally, some supporting cell types in the spleen appear to be different from those in the bone marrow. For instance, a decrease in NK cells in the spleen was associated with increased myeloid progenitors, suggesting that NK cells negatively regulate splenic hematopoiesis. However, some studies support the notion that T cells in the spleen act as hematopoietic niche cells^121,122^. Finally, macrophages play a role in supporting erythropoiesis and hematopoiesis in the spleen^123–126^. Taken together, what is known about EMH suggests that certain core factors are required for hematopoiesis in any organ and that there are organ-specific cell types or factors that can modulate these core processes.

## Cytokines with EMH potential

Below, this article provides a detailed look at individual cytokines with the potential to induce hematopoiesis and attempts to shed light on the mechanisms underlying the interaction between cancer and hematopoiesis.

## KIT ligand

KIT ligand is a fundamental hematopoietic growth factor and ligand of the receptor c-KIT^127,128^. KIT ligand has soluble and transmembrane forms^129^. Mice that lack function in both forms have severe or fatal anemia due to hematopoietic failure and pigment and germ cell deficiencies^130^. Mice lacking only the transmembrane form were still anemic, lacked pigmentation in the coat and were sterile^129^. Furthermore, the low body weight, anemia, and bone marrow cellularity in mice lacking transmembrane KIT ligands could not be mitigated by the overexpression of soluble KIT ligands but could be ameliorated with membrane-restricted KIT ligands^131^. Conversely, overexpressing membrane-restricted KIT ligands did not rescue reduced bone marrow myeloid progenitors in mice lacking transmembrane KIT ligands, but the overexpression of soluble KIT did^131^^,^ and the number of total peripheral blood leukocytes were rescued by both^131^. Finally, compared to wild-type mice, mice expressing KIT ligand lacking the main cleavage site, and therefore lacking the majority of soluble KIT ligands, did not exhibit differences in the number of hematopoietic progenitors in the bone marrow or in mature cells in the blood^132^.

The binding of KIT ligand to c-KIT dimerizes the receptor and activates its intrinsic tyrosine kinase activity^133–135^. The signaling characteristics vary between the different KIT ligand forms. Soluble KIT ligand signals rapidly and transiently followed by receptor degradation, while the membrane-associated form has sustained signaling^135^. Compared to soluble KIT ligands, membrane-associated KIT ligands induce longer-lasting downstream ERK1/2 and MAP kinase activity^136^. The inability of c-KIT to be internalized when bound to a membrane-associated KIT ligand may cause its sustained signaling, as immobilized anti-c-KIT antibodies also exhibit sustained signaling^137^. These data indicate that both forms of the KIT ligand are unique and functionally important and that the transmembrane form may play more important roles. Despite the importance of the c-KIT/KIT ligand for maintaining hematopoiesis, c-KIT signaling is also implicated in the quiescence of HSCs^138^. Additionally, c-KIT has been found to enhance the signaling of the EPO receptor and the IL-7 receptor, two important hematopoietic cytokines, as well as PDGFRα^139–141^. The KIT ligand and its receptor c-KIT clearly play crucial roles in hematopoiesis and have biological benefits befitting its centrality. The expression of c-Kit is likely to be a critical player in EMH, and studies investigating its regulation in the context of EMH are needed.

## CXCL12

CXCL12 is a pleotropic chemokine that plays various roles in development, hematopoiesis, inflammation, and injury repair. In hematopoiesis, CXCL12 is considered the major cytokine produced by the stem cell niche to retain HSPCs in the niche^142,143^. CXCL12 belongs to the CXC family of chemokines. Structurally, CXC family members have two conserved N-terminal cysteine residues that are separated by one variable residue, and these chemokines signal primarily through GPCRs^144,145^ The receptor predominantly recognized for binding CXCL12 is CXCR4, while binding to ACKR3 also occurs^146–150^. CXCR4 knockout animals die perinatally due to a combination of hematopoietic, neurogenic, vascular, and cardiogenic defects^151^. In contrast, ACKR3 knockout mice still die perinatally but exhibit normal hematopoiesis^152,153^. Signaling through CXCR4 is complicated and involves the activation of various Gα proteins, leading to the activation of the MAPK, PLC, and PI_3_K pathways^154,155^. Additionally, G protein-independent signaling pathways were identified. Although JAK-STAT signaling in CXCL12 cells has been reported^156–158^, other evidence challenges the importance of this pathway^159^. Activation of signaling downstream of beta-arrestin has also been reported^160–162^. CXCL12 and its receptor CXCR4 are uniquely functionally related in the context of hematopoiesis. However, the contribution of CXCL12 to the induction and maintenance of EMH is currently unknown, and given its role in maintaining hematopoiesis in the bone marrow, CXCL12 may even be a counterregulatory cytokine to EMH.

## Granulocyte colony-stimulating factor

Granulocyte colony-stimulating factor (G-CSF) is a cytokine that has potent hematopoiesis activity^163^. G-CSF signals through the homodimeric receptor G-CSFR^164^. Downstream of receptor activation is signaling by the JAK-STAT pathway, particularly through STAT3 and STAT5, which both promote proliferation, while STAT3 promotes differentiation^165^. G-CSF is thought to be produced primarily by stromal cells, such as fibroblasts and bone marrow niche cells, and by activated myeloid cells^166^. G-CSF levels are low during homeostasis but can increase during an immune response and subsequently decrease to baseline^167,168^. Mice lacking either G-CSF or its receptor have severe neutropenia but still produce small amounts of neutrophils^37,169^. G-CSF is also able to mobilize HSCs into circulation and establish EMH^18,170^.

## IL-1α and IL-1β

The IL-1 cytokine superfamily has 11 members corresponding to 10 different receptors. Within this cytokine family, there are three subfamilies: IL-1, IL-18, and IL-36. The unifying features of cytokines in this family include their lack of a signal peptide for secretion, cytoplasmic distribution as precursor molecules and the presence of a β-trefoil pyramidal barrel structure composed of six two-stranded β hairpins^171,172^. However, IL-1 receptor antagonist differs from the other family members regarding these shared features, as it lacks all of them. IL-1 signaling is transmitted through a trimeric complex comprised of the IL-1 receptor, the IL-1 family cytokines, and a coreceptor. When cytokines bind to primary receptors, they recruit coreceptors, and signaling can occur on the cytoplasmic side. For receptors with Toll-IL-1 receptor domains, MyD88 and downstream NF-κB signaling are activated. Additionally, the coreceptor can be present in a soluble form, either cleaved from the cell surface or produced by the liver. Both IL-1α and IL-1β signal through the IL-1R1 receptor and the coreceptor IL-1R3, both of which have Toll-IL-1 receptor domains. IL-1β binds to IL-1R2 with IL-1R3 as its coreceptor. IL-1β is a prototypical IL-1 family member. It is a very potent inflammatory molecule that plays a role in numerous diseases, including atherosclerosis and cancer^173,174^. IL-1β transcripts are induced by TLR ligands and by IL-1 itself. Following translation, IL-1β is in an inactive form until it is cleaved intracellularly by proteases such as the inflammasome or extracellularly by proteases such as proteinase 3, elastase, MMP-9, granzyme A, and mast cell chymase^171,175^. When recognized by HSCs, IL-1β stimulates proliferation and myelopoiesis^39^. IL-1α is considered a classic damage-associated molecular pattern that can initiate immune responses, and its biology reflects this process^176,177^. IL-1α is an unusual member of the IL-1 family because it is constitutively present in epithelial and mesenchymal cell types and does not require proteolysis for activation^171^. When proteolytically processed from its pro-form by proteases such as granzyme B, IL-1α becomes up to 10 times more potent^178^. IL-1α also has a nuclear localization signal, and when apoptosis occurs, IL-1α traffics to the nucleus, binds to chromatin, and becomes immunologically silent. In contrast, when necrosis occurs, IL-1α migrates to the cytoplasm, where it becomes immunologically active after the cell dies^179^. Given its unique biological activity, it is not surprising that IL-1α has been reported to contribute to autoimmune disease, microbial infections, and cancer^177^. IL-1 signaling has recently been shown to be associated with inflammatory changes in the bone marrow niche in that impair hematopoietic function during aging^108^. We recently showed that IL-1α induces TNFα expression in splenic HSPCs, which subsequently activates splenic niche activity^24^. In the present study, peripheral neutrophils were decreased in tumor-bearing mice treated with an IL-1R-blocking antibody. Overall, the unique biology of IL-1β and IL-1α results in potent initiators of nonsterile and sterile inflammation, respectively, suggesting that these family members might be potent initiators of EMH.

## Leukemia inhibitory factor

Leukemia inhibitory factor (LIF) is an IL-6 family member that signals through gp130 and the LIF receptor^180,181^. Signaling through the LIF receptor is also shared with other IL-6 family members, including OSM, CTNF, CT-1, and CLC, although all of these cytokines have additional receptors or coreceptors for signaling^180,182^. Signaling downstream of LIFR:gp130 is thought to be most prominent through JAK1, although signals can be transmitted through JAK2 and TYK2 as well^183–186^. JAK1 activation leads to the activation of STAT3, MAP kinase pathways, and PI_3_K in amounts that appear to be cell type-specific^187–189^. LIF is best known for its ability to maintain mouse embryonic stem cells in vitro^180^. STAT3 and PI3 kinase both lead to enhanced self-renewal and inhibition of differentiation in mouse embryonic stem cells, while MAP kinase activity activates differentiation^190–193^. LIF has several interesting functions outside of embryonic stem cells. Mutations in LIF have been reported in infertile women, and these reports concur with the failure of blastocyst implantation in LIF knockout dams and with the observation of high LIF expression in the endometrial glands^194–196^. In neuronal and stromal cell types, LIF also seems to increase growth while altering differentiation. Overexpression of LIF in an injected cell line led to bone marrow fibrosis and elevated osteoblast numbers^197^. Similarly, osteoblasts express the LIF receptor, and LIF enhances osteoblast differentiation while inhibiting adipocyte differentiation^198^. LIF overexpression by adenovirus injection enhances neural stem cell self-renewal, induces astrocyte differentiation in culture, and stimulates the proliferation of oligodendrocyte precursor cells when overexpressed by adenovirus^199–201^. In vitro-stimulated myoblasts exhibit increased proliferation but did not differentiate into myotubes, while LIF enhances muscle injury recovery in vivo^202,203^. In cancer, LIF expression has been reported in many solid tumors, such as colorectal, nasopharyngeal, skin, and breast cancer, and has been reported to support a variety of tumor functions^180,204^. Although the exploration of the role of LIF is ongoing, several lines of evidence suggest that LIF is a potent cytokine that impacts the proliferation and differentiation of stromal cells throughout the body. We recently reported that LIF is critical for the proliferation of cells localized in the EMH niche^24^, highlighting how inflammatory pathologies may interact with stromal components to regulate EMH during disease.

We believe that additional stromal active cytokines beyond LIF may also play important roles in EMH. Oncostatin M is an IL-6 family cytokine related to LIF that can signal through the LIF receptor and its own oncostatin M receptor^205^. Oncostatin M has been shown to promote myelopoiesis and to be active on stromal cells^206,207^. However, oncostatin M differs from LIF in important ways. Most importantly, oncostatin M can signal through STAT1 in addition to STAT3^205^. In regard to clinical disease, oncostatin M has been widely associated with joint disease and has been found in the synovial fluid of patients with rheumatoid arthritis^208–210^. Additionally, oncostatin M was recently identified as a biomarker of failure to respond to anti-TNFα therapy in patients with inflammatory bowel disease^211^. Taken together, these data indicate that oncostatin M is an important but understudied stromal active cytokine that is associated with human pathology and expanded hematopoiesis and has overlapping yet distinct effects compared with those of LIF.

## Tumor necrosis factor alpha

Tumor necrosis factor alpha (TNFα) is a quintessential inflammatory cytokine with wide-ranging and pleiotropic functions and effects^212^. TNFα is a trimeric member of the TNF family of cytokines. TNFα is produced as a membrane bound protein, primarily by immune cells such as monocytes and macrophages, that can be released as a soluble factor by the action of a specific protease, TACE or TNFα-converting enzyme^213^. TNFα can signal through two receptors, TNFR1 and TNFR2. The downstream signaling pathway of TNFα is complicated and involves several signaling mediators. The outcomes of TNFα signaling are highly context-dependent but can include NF-κB activation, MAPK activation, and cell death. In the context of cancer, TNFα plays roles in nearly every stage, including tumorigenesis, tumor growth, angiogenesis, and metastasis but also has anti-oncogenic effects and can be used as an anticancer treatment^212^. At the intersection of cancer and hematopoiesis, TNFα has been implicated in inducing myelopoiesis in the context of cancer^214^. With this link to myelopoiesis, local TNFα has also been shown to potentially be linked to EMH in the context of cancer.

## CXCR2 ligands

CXCR2 is a GPCR chemokine receptor with a wide repertoire of ligands that are highly conserved among vertebrates^215^. CXCR2 shares 77% amino acid identity with CXCR1 in humans and is positioned nearby on the same chromosome^216^. Ligands that bind to CXCR2 share a glutamic acid-leucine-arginine (ELR) motif and include CXCL1-3 and CXCL5-8^216^. CXCR2 is classically expressed on neutrophils, including G-MDSCs, and oligodendrocytes, although a wide selection of cells has been reported^215^. Upon activation of the receptor and after initiation of GPCR signaling, intracellular calcium stores are released into the cytoplasm, and gradient chemotaxis, degranulation, and MAPK activation can occur^217,218^. In the context of cancer, CXCL1-3 has been found to be directly expressed on human melanoma cells, and CXCL8 has been found to be produced by human prostate cancer cells^219,220^. Immune cells within the TME, such as macrophages, have also been shown to recruit G-MDSCs through CXCL2 expression^60^. Although not fully understood, CXCR2 ligands can mobilize HSPCs from the bone marrow despite the apparent lack of CXCR2 expression by these cells^221^. This ability of CXCR2 ligands to mobilize stem cells may be an important contributor to the induction of EMH.

## Concluding remarks and future perspectives

While hematopoiesis is essential for maintaining immune system function, it is also increasingly recognized as an important contributor to organismal pathology. Here, we have provided an overview of the interaction between solid tumors and hematopoiesis through extramedullary production of an immunosuppressive granulocyte subset and the systemic and niche factors enabling that production. Although this is still an emerging area of interest, the study of EMH holds promise for providing a better understanding of the alterations in hematopoiesis that occur in cancer and for better therapeutic approaches to cancer.

In this review, we present studies contributing to a broad understanding of the initiation and maintenance of EMH. Initially, solid tumors or noncancerous TME cells begin to produce CXCR2, either endogenously or through inflammation. These CXCR2 ligands recruit neutrophils to tumors while mobilizing HSPCs from the bone marrow. Cytokines are released simultaneously by the tumor and the TME induces the stromal cells in extramedullary sites to initiate hematopoiesis. Due to the unique biology of the extramedullary niche, circulating cytokines, or both, hematopoiesis in the extramedullary site becomes skewed toward the production of immunosuppressive granulocytes, G-MDSCs. These G-MDSCs can return to the TME via CXCR2 and support tumor growth. This broad overview is consistent with the available data and provides multiple avenues for therapeutic development and further exploration.

In addition to cancer, G-MDSCs have been found to be involved in many chronic inflammatory pathologies, suggesting that the processes underlying EMH may operate more broadly than previously appreciated^222^. G-MDSCs have been identified in human patients with viral and bacterial sepsis, where they are thought to efficiently resolve the infection^223,224^. Unlike their role in cancer, some studies have theorized that G-MDSCs promote disease resolution in the proper context. In rheumatoid arthritis, G-MDSCs have been identified in the synovial fluid of humans and mouse models, where their immunosuppressive function may be beneficial^225^. A study of dextran sulfate sodium (DSS)-induced colitis showed that G-MDSCs improved recovery when transplanted into mice just before the DSS model was established^226^. G-MDSC administration was also able to ameliorate experimental autoimmune encephalitis (EAE) in mice^227^. Another study revealed that multiple sclerosis patients with active disease had functional G-MDSCs^227^. The diversity of pathologies and both the beneficial and detrimental effects of G-MDSCs in various diseases suggest that controlling the induction and elimination of EMH is a potent target for therapeutic research in a broad range of clinically relevant inflammatory diseases.

The data suggest that additional extramedullary sites outside the liver and spleen deserve study. In the case of solid tumors, several unexpected places, such as the paraspinal region, peritoneum, bronchia, adrenal gland, endometrium, pancreas, and ureter, were also found to have EMH activity^11,13^. The breadth of the sites capable of conducting hematopoiesis is surprising and hints at a broader relevance of this phenomenon than is commonly appreciated. Additionally, as has been discussed in the literature, EMH is likely to be underreported because it does not produce distinguishing features on imaging and requires biopsy for confirmation. Moreover, clinicians are not often aware of the possibility of hematopoiesis occurring outside of the bone marrow and therefore may not be looking for it when treating patients^228^. Lymph node hematopoiesis is of interest because evidence suggests that these cells are the predominant site of EMH in cancer patients^11^. Conceptually, the lymph nodes and spleen share a function as organs of immune surveillance for different fluid compartments in the body, the lymph and blood, respectively. Additionally, on a more detailed level, the microanatomical organization of the two organs also rhymes. However, one key difference in the context of solid tumors is that the lymph node exhibits an increased load of cytokines from the tumor mass. Assuming that the lymph node stroma is as reactive as the spleen is, one would expect that lymph node hematopoiesis would be more dramatic than in the spleen itself. However, draining lymph nodes are also common primary sites of metastasis. Therefore, tumor growth in the lymph node may obscure any ongoing hematopoiesis.

The generation of treatments that manipulate the immune system to favor anticancer immunity has been an exciting development in modern cancer therapy. Central to the understanding of anticancer immune activation is that mutations in the cancer genome can produce novel antigens recognized by the adaptive immune system and reduce the expression of immune surveillance genes, leading to NK cell activation^229,230^. Procancer immune suppression is mediated by intrinsic mechanisms preventing chronic immune activation and cancer-induced immune cell phenotypic skewing away from activation^229^. These competing signals set a balance that is now being manipulated by therapeutics such as immune checkpoint inhibitors, recombinant cytokine therapy, and chimeric antigen receptor T-cell and NK-cell therapies^229,231–233^. However, care must be taken as the overactivation of the immune system via these therapies can lead to severe side effects^234,235^. Therapeutics developed with an understanding of cancer–immune interactions have been revolutionary, but progress still remains in terms of improving efficacy and minimizing side effects. Central to improving the efficacy of immune-targeting therapeutics is local activation of the immune system, a state modulated by systemically derived myeloid cells produced by EMH.

In conclusion, emerging studies on the initiation and maintenance of EMH have highlighted how cellular products from this process alter the course of clinically important diseases such as cancer. We look forward to future studies reporting the components of the extracellular niche and to clinical studies broadening the relevance of this pathological process to other disease states.
